# Infection control and the prevalence, management and outcomes of SARS-CoV-2 infections in mental health wards in London, UK: lessons learned from wave 1 to wave 2

**DOI:** 10.1192/bjo.2022.31

**Published:** 2022-03-08

**Authors:** Kathy Y. Liu, Anita Kulatilake, Chris Kalafatis, Gareth Smith, Jacob D. King, Jordi Serra-Mestres, Lauren Huzzey, Nicola Ng, Pooja Kandangwa, Thomas Elliott, Andrew Sommerlad, Louise Marston, Gill Livingston

**Affiliations:** Division of Psychiatry, University College London, UK; Central and North West London NHS Foundation Trust, UK; South London and Maudsley NHS Foundation Trust, and Department of Old Age Psychiatry, King's College London, UK; East London NHS Foundation Trust, UK; Central and North West London NHS Foundation Trust, UK; Central and North West London NHS Foundation Trust, UK; Barnet, Enfield and Haringey NHS Mental Health Trust, UK; Central and North West London NHS Foundation Trust, UK; Department of Old Age Psychiatry, King's College London, UK; Camden & Islington NHS Foundation Trust, UK; Division of Psychiatry, University College London, and Camden & Islington NHS Foundation Trust, UK; Department of Primary Care and Population Health, University College London; Division of Psychiatry, University College London, and Camden & Islington NHS Foundation Trust, UK

**Keywords:** Dementia, patients, in-patient treatment, COVID-19, older adults

## Abstract

**Background:**

Severe acute respiratory syndrome coronavirus 2 (SARS-CoV-2) disease (COVID-19) has high morbidity and mortality in older adults and people with dementia. Infection control and prevention measures potentially reduce transmission within hospitals.

**Aims:**

We aimed to replicate our earlier study of London mental health in-patients to examine changes in clinical guidance and practice and associated COVID-19 prevalence and outcomes between COVID-19 waves 1 and 2 (1 March to 30 April 2020 and 14 December 2020 to 15 February 2021).

**Method:**

We collected the 2 month period prevalence of wave 2 of COVID-19 in older (≥65 years) in-patients and those with dementia, as well as patients’ characteristics, management and outcomes, including vaccinations. We compared these results with those of our wave 1 study.

**Results:**

Sites reported that routine testing and personal protective equipment were available, and routine patient isolation on admission occurred throughout wave 2. COVID-19 infection occurred in 91/358 (25%; 95% CI 21–30%) *v*. 131/344, (38%; 95% CI 33–43%) *P* < 0.001 in wave 1. Hospitals identified more asymptomatic carriers (26/91; 29% *v*. 16/130; 12%) and fewer deaths (12/91; 13% *v*. 19/131; 15%; odds ratio = 0.92; 0.37–1.81) compared with wave 1. The patient vaccination uptake rate was 49/58 (85%).

**Conclusions:**

Patients in psychiatric in-patient settings, mostly admitted without known SARS-CoV-2 infection, had a high risk of infection compared with people in the community but lower than that during wave 1. Availability of infection control measures in line with a policy of parity of esteem between mental and physical health appears to have lowered within-hospital COVID-19 infections and deaths. Cautious management of vulnerable patient groups including mental health patients may reduce the future impact of COVID-19.

Older adults and people with dementia have high rates of infection and mortality due to severe acute respiratory syndrome coronavirus 2 (SARS-CoV-2) disease (COVID-19).^1,2^ In England, three national pandemic lockdowns commenced on 23 March 2020, 5 November 2020 and January 6 2021 during two pandemic waves estimated to occur between March and May 2020 (peak in March–April) and September 2020 to April 2021 (two peaks in November and December 2020).^3^ National guidance to prevent and control COVID-19 infections within health and care settings was introduced during this period,^4^ aiming to reduce the risk of outbreaks. From 27 April 2020, National Health Service (NHS) hospitals expanded COVID-19 testing to all patients, including asymptomatic patients.^5^ From 15 June 2020, all healthcare staff and visitors were expected to wear surgical masks in all hospital areas,^6^ and from 16 November 2020, regular testing of asymptomatic patient-facing NHS staff was advised.^7^

We previously examined psychiatric hospital in-patients who were over 65 years old or had dementia in London, UK, during the first wave of the COVID-19 pandemic between 1 March and 30 April 2020. We found a higher rate of SARS-CoV-2 infection (38% of *N* = 344) and a higher proportion of deaths due to COVID-19 (15% of *N* = 131) in our sample compared with reported community rates.^8^ Delayed access to personal protective equipment (PPE) and SARS-CoV-2 polymerase chain reaction (PCR) tests probably contributed to the high infection rate. We recommended for future waves that: (a) all patients should be screened for COVID-19 at the time of admission and, in suspected cases, isolated until the diagnosis is known; (b) re-testing should be offered during admission; (c) PPE should be easily available; and (d) symptomatic staff should be offered SARS-CoV-2 tests. These measures have the potential to reduce COVID-19 transmission in a vulnerable patient group. One study showed that infection prevention measures introduced on an in-patient geriatric psychiatry unit in the USA contained a SARS-CoV-2 outbreak over the subsequent 2 months,^9^ but the sample size was relatively small (19/27 patients had confirmed SARS-CoV-2 infection). Another study showed that improvements in infection control and prevention measures in a mental health unit in Chengdu, China,^10^ led to no COVID-19 cases, although this was in the context of very low regional COVID-19 prevalence during the study period.^11^

We therefore aimed to replicate our earlier study^8^ in 16 psychiatric wards to examine changes in clinical guidance, practice and outcome between the first and second COVID-19 waves in England. We collected data for older (≥65 years) in-patients and those with dementia in the same five NHS mental health trusts during the second wave of the COVID-19 pandemic in London, UK (14 December 2020 to 15 February 2021) to assess whether there were any differences in the management and treatment of SARS-CoV-2 infections, and, if so, whether these were accompanied by lower COVID-19 prevalence and better outcomes.

## Method

### Study design and participants

The study design and inclusion criteria were the same as in the earlier study.^8^ We included all older people (aged ≥65 years) and people with young-onset dementia (with no age restrictions) who were psychiatric in-patients and diagnosed with COVID-19 between 14 December 2020 and 15 February 2021. Participants were either admitted during the study period or were existing patients at the start of the study at one of the same five participating London NHS mental health trusts: Camden and Islington NHS Foundation Trust, East London NHS Foundation Trust, South London and Maudsley NHS Foundation Trust, Central and North West London NHS Foundation Trust, and Barnet, Enfield and Haringey Mental Health NHS Trust. These trusts cover 14 boroughs in inner and outer Greater London with around 4.6 million ethnically diverse residents, or 51% of London's and 7% of the UK's population.

We obtained Health Research Authority and Health and Care Research Wales approval and ethical approval (IRAS 284782) from West Midlands – Coventry and Warwickshire Research Ethics Committee (reference 20/WM/0165). In the UK, the health and social care system is taking action to manage and mitigate the spread and impact of the current outbreak of COVID-19; therefore, our ethics approval allowed study clinicians to gather anonymised data retrospectively in their services without asking individual patients to consent.

### Data collection

There were 16 wards in the five trusts. Clinicians within the study sites recorded anonymised data retrospectively from electronic patient records using a standardised electronic data form for all patients who had confirmed COVID-19 and clinical details about each site. The data were collected between 7 April and 7 June 2021, and up-to-date vaccination and outcome data were obtained. We collected the same demographic, physical and mental health, Mental Health Act 1983 or Mental Capacity Act 2005 status (laws around detaining and treating those with mental illness) and COVID-19 related data as reported in the earlier study.^8^ We asked about site visitor policy, the types of SARS-CoV-2 test (lateral flow or PCR tests) that were offered, and the availability and frequency of tests for hospital staff. We collected additional data on COVID-19 admission screening tests, routine follow-up tests, and vaccination offer and uptake. In August 2020, Public Health England changed its definition of death due to COVID-19 to a death in a person with a laboratory-confirmed COVID-19 test who died within (equal to or less than) 28 days of the first positive specimen date,^12^ so we included this outcome measure in the current study.

### Statistical analysis

As in our earlier study, we described the 2 month period prevalence of COVID-19, as well as characteristics of patients with COVID-19 and their management and outcomes. Differences in means and proportions between the two study periods were assessed using a two-sample *t*-test or χ-squared test, respectively, with significance level ɑ = 0.05. Missing data points were excluded from analyses. To compare deaths between the study periods, we performed a logistic regression to assess whether admission during the second study period influenced the probability (odds ratio [OR]) of death at any time compared with the first study period, after controlling for age (the earlier study was the reference group).

## Results

### Characteristics of COVID-19 positive patients

Sociodemographic, medical and psychiatric characteristics of in-patients diagnosed with COVID-19 (Table 1) were similar across the two study periods, although a lower proportion of in-patients in the second study period (December 2020 to February 2021) were legally detained within the Mental Capacity Act Deprivation of Liberty Safeguards.

**Table 1:** Sociodemographic and medical/psychiatric characteristics of patients diagnosed with COVID-19 during the study period

COVID-19-positive in-patients	December 2020 to February 2021 (*N* = 91)	
Age, years	Mean (s.d.)	77.0 (7.2)
Age group	51–64	2 (2%)
	65–80	61 (67%)
	81–95	28 (31%)
Sex	Female	52 (57%)
Ethnicity	White	63/89 (71%)
	Black Caribbean, African or British	17/89 (19%)
	Asian or Asian British	6/89 (7%)
	Mixed or Other	3/89 (3%)
Dementia status	Young-onset dementia	5 (5%)
	Late-onset dementia	41 (45%)
	No dementia	45 (49%)
Legal status on entry	Informal	25 (27%)
	Mental Health Act Section 2	40 (44%)
	Mental Health Act Section 3	16 (18%)
	Mental Capacity Act Deprivation of Liberty Safeguards	10 (11%)
Comorbidities	Hypertension	42 (46%)
	Chronic cardiac disease	26 (29%)
	Any diabetes	22 (24%)
	Chronic kidney disease	16/90 (18%)
	Chronic obstructive pulmonary disease	17 (19%)
	Asthma	10 (11%)
	Active cancer	7 (8%)
Body mass index	<18.5	15/81 (19%)
	18.5 to 25	40/81 (49%)
	25 to 30	16/81 (20%)
	≥30	10/81 (12)
Smoking	Not a current smoker	79/86 (92%)
	Current smoker	7/86 (8%)

### COVID-19 period prevalence

Between 14 December 2020 and 15 February 2021, the overall period prevalence of COVID-19 in the sample population was 25% (95% CI 21–30%), which was significantly lower than the period prevalence between 1 March and 30 April 2020 (131/344 (25%) *v*. 91/358 (38%) *P* < 0.001) (Table 2). Of 91 patients who tested positive during the study period, five (5%) were known to have had a previous positive COVID-19 PCR test.

**Table 2:** Number of in-patients and COVID-19 period prevalence across the five trusts during the two study periods

All in-patients	Study period	
	December 2020 to February 2021 (*N* = 358)	March to April 2020 (*N* = 344)
Total number of in-patients during the study period (range across trusts)	358 (33–105)	344 (33–92)
Total number of in-patients diagnosed with COVID-19	91	131
Patients diagnosed with COVID-19 who were existing patients at the start of study period	60 (66%)	89 (68%)
Patients diagnosed with COVID-19 who were admitted during the study period	31 (34%)	42 (32%)
Period prevalence of COVID-19 (95% CI)***	91/358; 25% (21–30%)	131/344; 38% (33–43%)
Range of period prevalence of COVID-19 across trusts (*N*)	19% (20/105) to 32% (26/81)	29% (27/92) to 52% (45/86)

***P < 0.001

### Site infection control and prevention measures

During March to April 2020, there was an average delay across sites of 4.5 days and up to 7 days from the first suspected COVID-19 case to the availability of SARS-CoV-2 PCR tests and PPE, respectively.^8^ By contrast, during the current study period (December 2020 to February 2021), PCR tests and PPE were reported to be available in all sites throughout the study period. Routine screening of patients for COVID-19 prior to admission took place on all sites for patients transferred from other hospitals, but patients were admitted from the community without prior testing. Four out of five sites required PCR tests for pre-admission screening, and one accepted lateral flow tests. All sites routinely offered weekly PCR tests to patients who were not suspected of having COVID-19 during admission. Two sites also offered re-testing on days 1, 3 and 5 of admission prior to weekly testing. All sites reported that asymptomatic staff were offered or required to take regular COVID-19 tests (lateral flow or PCR) 1–2 times per week. Fifteen of the 16 wards had oxygen on the ward for treatment during the study period. Some sites reported receiving educational input (e.g. webinars), having direct phone access to senior physicians, virtual ward rounds or palliative care liaison. No wards closed to admissions during the study period, and two sites had a designated COVID-19 ward in the mental health unit where COVID-19 positive patients were transferred. Visitors were either not allowed on the wards, or allowed in exceptional circumstances or for specific meetings with healthcare professionals.

Of all new admissions who tested positive for COVID-19 (*N* = 31/127, 24%), the median number of days between admission and the first COVID-19 screening (PCR) test related to the admission was 0 (interquartile range [IQR] 0–1) days. The result of the screening test was positive in eight (26% of *N* = 31 or 6% of *N* = 127) patients. All new patients admitted during the study period were isolated on admission. Most (30/31, 97%) new admissions were subsequently offered a routine follow-up test, with a median of 8 (IQR 3–13) days between the screening and the first routine follow-up COVID-19 test. This was positive in 12/30 (40%). Among new admissions and existing in-patients, a routine follow-up PCR test was offered to 85/91 (93%) patients during the study period, and 23/83 (28%) patients became positive at the earliest routine follow-up test.

### COVID-19 vaccinations

One patient was known to have received a first vaccine dose prior to their admission. Of the other 89 patients (one had missing data), 58 (65%) were offered a first vaccine dose and 49 (55%) received this during their admission, equating to an 85% vaccine acceptance rate. All first vaccination doses were between 14 January and 12 April 2021 for those who received the vaccine during their admission. The median duration from study start to first vaccination dose was 60 (IQR 52–66) days, equivalent to 12 February 2021.

### COVID-19 symptoms

Almost a third (29%) of patients in the current study were asymptomatic, which was a significantly higher proportion than our previous finding of 12% (*P* = 0.002). Most patients (76%) did not self-report symptoms (Table 3). Apart from a significantly lower proportion who had a raised temperature during the current study period, the symptom profile of symptomatic patients was similar between the two study periods, with the most commonly reported or detected symptoms being new persistent cough (51%), fatigue (40%), shortness of breath (38%) and loss of appetite (35%). Acute cognitive decline or delirium occurred almost as frequently as raised temperature >37.8 °C (35%) and affected a higher proportion of people with dementia versus those with no dementia.

**Table 3:** COVID-19 symptoms. Data from the earlier study period (March to April 2020) were obtained separately^8^

COVID-19 positive in-patients	Study period		*P*-value
	March to April 2020 (*N* = 131)	December 2020 to February 2021 (*N* = 91)	
*COVID-19 symptoms*			
Symptomatic	114/130 (88%)	65 (71%)	0.002
Patient self-reported symptoms	19/108 (18%)	16/63 (25%)	0.222
*Symptoms reported/detected*			
Temperature >37.8 °C	73/114 (64%)	20/58 (34%)	<0.001
New persistent cough	57/114 (50%)	33/65 (51%)	0.921
Shortness of breath	38/114 (33%)	25/65 (38%)	0.490
Gastrointestinal symptoms/diarrhoea/vomiting	19/114 (17%)	8/65 (12%)	0.433
Fatigue	56/112 (50%)	26/65 (40%)	0.198
Loss of appetite	41/114 (36%)	23/65 (35%)	0.938
Acute cognitive decline/delirium	44/114 (39%)	23/65 (35%)	0.669
Sore throat	4/114 (4%)	7/65 (11%)	0.052

### COVID-19 treatment and outcomes

The treatment of patients diagnosed with COVID-19 was similar across the two study periods, apart from a lower proportion of patients who received prophylactic antibiotics and a higher proportion who received venous thromboembolism prophylaxis in the current (December 2020 to February 2021) compared with the earlier study period (Table 4). Although there were numerically fewer deaths in the second study period, there was no significant difference in deaths (at any time) between the study periods after controlling for age (OR 0.82, 95% CI 0.37–1.81 for the second study versus the first study).

**Table 4:** COVID-19 treatment and outcome

COVID-19 positive in-patients	Study period		*P*-value
	March to April 2020 (*N* = 131)	December 2020 to February 2021 (*N* = 91)	
Vitamin D treatment	58/111 (52%)	37 (41%)	0.100
Prophylactic antibiotics for community-acquired or hospital-acquired pneumonia	54/127 (43%)	9 (10%)	<0.001
Therapeutic antibiotics for community-acquired or hospital-acquired pneumonia	−	31 (34%)	−
Venous thromboembolism prophylaxis	27/118 (23%)	33/81 (41%)	0.007
Oxygen therapy on the ward	27 (21%)	23 (25%)	0.413
Dexamethasone^a^	−	8 (9%)	
Remdesivir^a^	−	1 (1%)	
Transferred to a general hospital for COVID-19 treatment	42 (32%)	31/90 (34%)	0.711
*Outcome*			0.197
Recovered	111 (85%)	75 (82%)	
Died (at any time)	19 (15%)	12 (13%)	
Still unwell	1 (1%)	4 (4%)	
Died within 28 days of a positive COVID-19 test^b^	−	10 (11%)	

a. Treatment not available in March and April 2020.

b. Data not available in March and April 2020 because Public Health England changed its definition of death due to COVID-19 in August 2020 to a death in a person with a laboratory-confirmed COVID-19 test who died within (equal to or less than) 28 days.

## Discussion

In this study, we report detailed follow-up data on COVID-19 prevalence symptoms and outcomes in psychiatric in-patients aged over 65 years or who had dementia during the second COVID-19 wave in London, UK, from December 2020 to February 2021. In contrast to earlier findings in a similar patient population during the first wave from March to April 2020, we found that infection control measures such as admission screening and routine follow-up PCR tests, asymptomatic testing of staff, PPE availability, and isolation procedures were in place from the start of the study. These measures probably contributed to the identification of a higher proportion of asymptomatic carriers and, even with this higher proportion, to a lower period prevalence of COVID-19 infection (25%) and numerically fewer deaths within the psychiatric hospital sites during the current study period.

As in our earlier study, the COVID-19 period prevalence in the mental health units of 25% was higher than the reported community rate, which was up to 3.5% over 2 weeks within the study period in London (excluding care homes, hospitals and other institutional settings),^13^ although systematic screening of individuals did not take place in the community. We observed that 26% of new admissions who were COVID-19 positive tested positive on admission, which may be related to the finding that most of these patients (71%) came from the emergency department, general hospital, other mental health units or care homes, where COVID-19 prevalence was already relatively high. In addition to 60 existing in-patients who tested positive for COVID-19 during their admission over the study period (more than 7 days after admission), another 15 new patients admitted during the current study period may have contracted COVID-19 during their admission, as they were COVID-19 negative on admission and at the first routine follow-up but subsequently tested positive, equating to a within-hospital infection rate estimate of 21% (75 of 358). This is similar to the within-hospital infection rate (defined as patients who test positive for COVID-19 more than 7 days after admission) reported by NHS England of up to 19% during the same period.^14^ We may have expected rates to be lower in psychiatric hospitals where patients are not admitted for physical illnesses, including COVID-19. Although a number of infection control measures were in place nationally in hospitals during the study period,^4^ there was variability in applying these,^15^ with challenges including suboptimal ventilation, insufficient staffing levels, and shared computers, desks and rest facilities for staff. There may be specific difficulties in psychiatric settings related to managing patients with severe mental illness and dementia who cannot understand, remember and abide by mask-wearing and social distancing, and are ambulatory in a shared ward space.

Among patients who tested positive for COVID-19 during the current study period, medical management was similar to the earlier study period, except that a higher proportion received venous thromboembolism (VTE) prophylaxis and a lower proportion received prophylactic antibiotics for pneumonia, in line with national guidelines that recommend use of VTE prophylaxis and avoidance of antibiotics unless there is clinical suspicion of bacterial pneumonia.^16^ Although the proportion who died or remained unwell from COVID-19 did not differ significantly, the lower COVID-19 period prevalence meant that there were numerically fewer deaths, suggesting that infection control measures affected mortality outcomes through reducing overall infection prevalence rather than by reducing mortality rates in infected individuals.

As newly admitted patients during the study period were tested for COVID-19 on admission and all patients were routinely re-tested, this study is likely to provide a more accurate prevalence rate and description of the symptom profile of COVID-19 positive psychiatric in-patients aged over 65 years or those with dementia, compared to the earlier study period where testing was initially unavailable and not carried out routinely so we considered that we may have missed asymptomatic patients and underestimated the prevalence. In keeping with this, we found that a higher proportion (29%) of in-patients during the current study period was asymptomatic, in line with earlier studies in older adults and nursing home residents that reported the rate of asymptomatic infection to be as high as 36–39%.^17,18^ This reflects the availability of testing meaning that tests were not reserved for those with symptoms. Our finding that 35% of symptomatic in-patients experienced delirium is also consistent with but higher than a larger study of COVID-19-positive hospitalised older adults, 13% of whom had dementia, which found that delirium was present in 26% of the sample.^19^

The first dose vaccination was offered to 65% and accepted by 55% of psychiatric patients. The vaccine was not offered to all patients early despite their clear vulnerability, and the uptake rate (85%) was slightly lower compared to the London vaccination uptake rate in eligible care home residents between 8th December and 14th February (93%),^20^ and the national vaccination uptake rate (88–95%) in adults aged over 60 years on 12 April 2021,^21^ which was the latest date a first vaccine dose was received by any COVID-19 positive patient in the study. A slightly lower vaccine uptake rate in patients with mental health illness compared to the general population has previously been reported,^22^ but this was not a consistent finding.^23^ It is possible that treatment and isolation for COVID-19 delayed the administration of vaccines or meant that some patients were not eligible for vaccination, as Public Health England guidelines state that vaccinations should not be given until 28 days after a positive COVID-19 test.^24^ The NHS started administering COVID-19 vaccinations in England on 8 December 2020, and NHS England announced that all individuals within the Joint Committee on Vaccination and Immunisation (JCVI) priority groups 1–9, which included the study population, had been offered the vaccination by 12 April 2021.^25^ It would be important to investigate the prevalence and outcomes of COVID-19 in a future study population where most patients have been vaccinated, to monitor the impact of vaccination in this group.

Our study's strengths include a large target population covering over half the population of London and 7% of the UK, and methodology consistent with our previous study allowing comparison, but there are limitations. Although both study periods coincided with the first and second pandemic waves in England, suggesting that infection rates were likely to be comparable, we could not rule out the possibility that differences in community COVID-19 prevalence rates between the two study periods could have contributed to the lower hospital period prevalence during the current study period. This is because testing was not routinely available to the public during the first lockdown so community infection rates during this period were underestimated. Vaccination also occurred during wave 2 but not wave 1, but it is unclear to what extent this may have influenced differences in reported outcomes as the study period coincided with the beginning of the vaccination campaign. We did not record the prevalence of COVID-19 in hospital staff and there may also have been nosocomial transmission between healthcare staff and patients. All deaths in the first study, whether before or after 28 days, were within the two-month study period and attributed to COVID-19, but the change in the NHS England definition of deaths due to COVID-19 after the first study period limited our ability to compare the proportions of deaths using this updated figure, thus we compared deaths at any time. It was also not possible to directly compare the within-hospital infection rates between the two study periods, as although the infection rate during the previous study period (38%) appears to mainly represent within-hospital infections, as no patients were known or suspected to have COVID-19 on admission (the first clinically suspected COVID-19 case was two weeks after the study start date), the lack of initial routine testing during this period means that the detection of new asymptomatic admissions may have been missed. It has been estimated that the hospital-acquired infection proportion in UK mental health hospitals was 67.5% during the first pandemic wave.^26^ Clinicians gathered data from electronic patient records, which minimised the influence of recall bias but might have contained unknown relevant omissions or errors. We also considered that our modest sample size meant that we were unable to meaningfully explore whether admission to any individual ward influenced outcomes. Future larger studies should also explore whether specific major psychiatric disorders may influence outcomes in this population.

Overall, improvements in infection control measures on older adult psychiatric wards, including routine testing of patients and staff, isolation of patients on admission and availability of PPE, is likely to be related to a significant reduction in COVID-19 period prevalence and correspondingly fewer deaths in older adult in-patients and those with dementia. The availability and use of PPE and testing, consistent with a policy of parity of esteem between mental and physical health, appears to have occurred and reduced excess within hospital transmissions and number of deaths. However, the infection rate remains high, and this is clearly an at-risk population who are difficult to isolate. It may be difficult to offer vaccines to this vulnerable population, many of whom would not have capacity to consent or refuse or have understood why they were having it. However, they should be prioritised as those in care homes were, given similar high-risk profiles: age, likelihood of physical comorbidities and communal living. Routine and frequent testing is important as almost a third of COVID-19 positive patients were asymptomatic and an estimated 21% acquired COVID-19 during their admission. Admission from another hospital or care home may have increased the risk of having COVID-19 on admission. Future research should consider the impact of vaccination on future prevalence and outcomes, including symptoms, of COVID-19 in older adults and people with dementia. Although vaccination is likely to reduce serious illness from COVID-19,^27^ cautious management of vulnerable patient groups remains appropriate to reduce the impact of future COVID-19 waves.

## Data Availability

Because the data for study were sensitive and gathered without individual permission, they are not available for sharing.
